# Evaluation on antithrombotic effect of aspirin eugenol ester from the view of platelet aggregation, hemorheology, TXB_2_/6-keto-PGF_1α_ and blood biochemistry in rat model

**DOI:** 10.1186/s12917-016-0738-0

**Published:** 2016-06-14

**Authors:** Ning Ma, Xi-Wang Liu, Ya-Jun Yang, Dong-Shuai Shen, Xiao-Le Zhao, Isam Mohamed, Xiao-Jun Kong, Jian-Yong Li

**Affiliations:** Key Lab of New Animal Drug Project, Gansu Province Lanzhou, 730050 People’s Republic of China; Key Lab of Veterinary Pharmaceutical Development, Ministry of Agriculture, Lanzhou, 730050 People’s Republic of China; Lanzhou Institute of Husbandry and Pharmaceutical Science of CAAS, Lanzhou, 730050 People’s Republic of China; No.335, Jiangouyan, Qilihe District, Lanzhou, 730050 China

**Keywords:** Aspirin eugenol ester (AEE), Thrombosis, k-carrageenan, Rat, Platelet aggregation, Blood viscosity

## Abstract

**Background:**

Based on the prodrug principle, aspirin and eugenol, as starting precursors, were esterified to synthesize aspirin eugenol ester (AEE). The aim of the present study was to evaluate the antithrombotic effect of AEE in an animal disease model. In order to compare the therapeutic effects of AEE and its precursors, aspirin, eugenol and a combination of aspirin and eugenol were designed at the same molar quantities as the AEE medium dose in the control group.

**Methods:**

After oral administration of AEE (dosed at 18, 36 and 72 mg/kg) for seven days, rats were treated with k-carrageenan to induce tail thrombosis. Following the same method, aspirin (20 mg/kg), eugenol (18 mg/kg) and 0.5 % CMC-Na (30 mg/kg) were administered as control drug. Different drug effects on platelet aggregation, hemorheology, TXB_2_/6-keto-PGF_1α_ ratio and blood biochemistry were studied.

**Results:**

AEE significantly inhibited ADP and AA-induced platelet aggregation in vivo. AEE also significantly reduced blood and plasma viscosity. Moreover, AEE down-regulated TXB_2_ and up-regulated 6-keto-PGF_1α_, normalizing the TXB_2_/6-keto-PGF_1α_ ratio and blood biochemical profile. In comparison with aspirin and eugenol, AEE produced more positive therapeutic effects than its precursors under the same molar quantity.

**Conclusion:**

It may be concluded that AEE was a good candidate for new antithrombotic and antiplatelet medicine. Additionally, this study may help to understand how AEE works on antithrombosis in different ways.

## Background

In the last century, remarkable increases of life spans in dogs and cats have promoted the development of geriatric veterinary medicine [1, 2]. The findings have proved that age-related thrombin was a possible risk factor in dogs and cats [3, 4]. Thus, there is a need for anti-thrombotic drugs for animals. The physiological process of thrombosis is complex and influenced by many factors such as platelet aggregation, ratio of thromboxane B_2_ (TXB_2_) to 6-keto prostaglandin F_1α_ (6-keto-PGF_1α_), biochemical and hemorheological parameters [5–8]. Therefore, the impacts of drugs on platelet aggregation, hemorheology, TXB_2_/6-keto-PGF_1α_ ratio and blood biochemistry can be employed for examining the effect of compound in thrombosis prevention [9].

Aspirin is extensively used in human and veterinary medicine to reduce inflammation, pain and fever. In cats, aspirin is not well tolerated because of the deficient in glucuronate and has a prolonged elimination half-time. The pharmacodynamics of aspirin are similar in human and dogs. Related researches have proved that prophylactic use of aspirin can reduce the risk of heart attack, stroke and some cancers [10, 11]. A number of reports have showed that eugenol has antiseptic, analgesic, antibacterial, antiplatelet aggregation and anticancer properties [12–14]. Notably, aspirin and eugenol produce similar pharmacological effects especially on inflammation and platelet aggregation.

Gastrointestinal damage caused by aspirin and vulnerability of eugenol limit their application. These side effects and structural instability are related to chemically carboxyl group of aspirin and hydroxyl group of eugenol. In order to increase therapeutic effect and stabilization, aspirin and eugenol as starting precursors were esterified to synthesize aspirin eugenol ester (AEE). AEE, a pale yellow, odorless crystal, overcomes the disadvantages of its precursors. Anti-ulcerogenic activity of eugenol has been confirmed in a previous study, in which eugenol (100 mg/kg, p.o.) significantly and dose-dependently reduced gastric ulcers induced by platelet activating factor and ethanol in Wistar rats [15]. So a combination of eugenol and aspirin could play complementary roles to reduce gastrointestinal damage and enhance the therapeutic effects. A 15-day subchronic toxicity study identified that no histopathological changes was observed in any organ from the rats following low-dose AEE (50 mg/kg), whereas mucosa height of stomach, duodenum and ileum were increased in rats following high-dose AEE exposure (2000 mg/kg) [16].

Carrageenan-induced rat tail thrombosis model has been widely used to evaluate whether substances have antithrombotic effects during the drug discovery stage [17–20]. The length of thrombosis in rat tail directly reflects the effects of drug on thrombosis formation [21, 22].

The preventive effects of AEE on thrombosis formation have been confirmed in k-carrageenan-induced rat tail thrombosis model, in which AEE significantly inhibited thrombus formation and reduced the tail thrombosis length [23]. However, investigations of the preventive effects of AEE against thrombosis are not sufficient. In order to understand and illustrate the possible underlying mechanism of AEE, a follow-up study was carried out using the same animal disease model and drug dosage. The aim of the present study was to evaluate the effects of AEE on platelet aggregation, hemorheology, blood biochemistry and TXB_2_/6-keto-PGF_1α_ ratio. To compare the effects of AEE and its precursors, equimolar doses of aspirin, eugenol and combination of aspirin and eugenol were also administered in the experiment.

## Methods

### Chemicals and reagents

AEE (transparent crystal, 99.5 % purity with RP-HPLC) was prepared in Key Lab of New Animal Drug Project of Gansu Province, Key Lab of Veterinary Pharmaceutical Development of Agricultural Ministry, Lanzhou Institute of Husbandry and Pharmaceutical Sciences of CAAS. CMC-Na was supplied by Tianjin Chemical Reagent Company (Tianjin, China). Aspirin and Tween-80 were obtained from Aladdin Industrial Corporation (Shanghai China). Eugenol was supplied by Sinopharm Chemical Reagent Co., Ltd. (Shanghai China). Adenosine diphosphate (ADP), arachidonic acid (AA) and k-carrageenan were purchased from Sigma (St. Louis, USA). Blood biochemical analysis reagents were obtained from Meikang Co., Ltd (Zhejiang, China). All chemical reagents were of analytical reagent grade. A Thermo Fisher Scientific Multiskan Go 1510 was used for spectrophotometry (USA).

### Animals and treatment

Ninety male Wistar rats (approximately 160 g) were purchased from the animal breeding facilities of Lanzhou Army General Hospital (Lanzhou, China). Animals were housed in plastic cages (size: 50 × 35 × 20 cm, 10 rats per cage) with stainless steel wire cover and chopped bedding in a ventilated room. The light/dark regime was 12/12 h and living temperature was maintained at 22 ± 2 °C with relative humidity of 55 ± 10 %. The animals had free access to a standard diet and tap water (Standard compressed rat feed from Keao Xieli Co., Ltd., Beijing, China). Rats were allowed a 2-week acclimation period prior to the start of experiment.

### Drug preparation

AEE and aspirin suspensions were prepared in 0.5 % of CMC-Na. Eugenol and Tween-80 (mass ratio of 1:2) were mixed with distilled water. Kappa-carrageenan (k-carrageenan) was dissolved in physiological saline.

### Study design

Ninety rats were divided into 9 groups in the study. According to individual body weight (BW), rats were intragastrically (i.g.) administered with 18, 36 and 72 mg/kg AEE, 20 mg/kg aspirin or 18 mg/kg eugenol. For the comparability of the experimental results, medium-dose AEE, aspirin and eugenol were administered at the dose of 0.11 mmol. In order to compare AEE with its precursors, an equimolar combination of aspirin and eugenol was designed in the experiment. Rats in CMC-Na group were given 0.5 % CMC-Na at the dose of 30 mg/kg BW, and the volume of CMC-Na was nearly equal in comparison with other drug suspensions. A detailed description of the study design is shown in Table 1. All drugs and CMC-Na were administered intragastrically for seven days, after which the rats were intraperitoneally injected with 20 mg/kg k-carrageenan to induce thrombosis.

**Table 1:** Design of the experiment

Groups	Drug	Dosage (mg/kg)	Molar quantity (mmol)
Control	–	–	–
Model	–	–	–
CMC-Na	CMC-Na	30	–
Aspirin	aspirin	20	0.11
Eugenol	eugenol	18	0.11
AEE L	AEE	18	0.06
AEE M	AEE	36	0.11
AEE H	AEE	72	0.22
Combination	aspirin + eugenol	20 + 18	0.11

Ninety rats were divided into 9 groups (*n* = 10). CMC-Na was used as the vehicle. The volumes of CMC-Na and drug suspension were nearly equal. The molar quantity of medium-dose AEE, aspirin and eugenol are same at 0.11 mmol. An equimolar combination of aspirin and eugenol was used in the study. Rats in the model, CMC-Na, aspirin, eugenol, AEE and combination groups were intraperitoneally injected with 20 mg/kg k-carrageenan to induce thrombosis

### Carrageenan-induced rat tail thrombosis model

Carrageenan-induced rat tail thrombosis was induced as described in the previous study [23].

### Measurement of rat tail thrombosis length

After the last treatment with different drugs, each rat was intraperitoneally injected with k-carrageenan. Swelling and redness in rat tail were monitored. Thrombus lengths were measured and photographed at 24 and 48 h.

### Blood sampling

After the last measurement of thrombosis length at 48 h, rats were anesthetized with 10 % chloral hydrate and blood samples were taken from the heart. According to the requirement of the experiment, blood samples were collected in different volume with different anticoagulants. Serum for biochemical analysis and plasma for ELISA were stored at −80 °C until the day of analysis. All platelet aggregation and hemorheological tests were performed within 3 h of blood collection.

### Hemorheological tests

Blood samples of approximately 2 mL were collected and anticoagulated with EDTA-K_2_, after which 0.8 mL whole blood samples were prepared for whole blood viscosity examination. The remaining blood samples were centrifuged at 1000 g for 10 min to obtain plasma, after which 0.5 mL of plasma was used for plasma viscosity examination. A kone plate viscometer LBY-N6A analytical instrument (Precil Company, Beijing, China) was used for the measurement of whole blood viscosity and plasma viscosity at 37.0 ± 0.5 °C. For whole blood viscosity, measurements were made at three shear rates: 5, 100 and 200 s^−1^. Plasma viscosity was measured at a shear rate of 100 s^−1^.

### Assay of platelet aggregation

In vivo assessment of the anti-platelet aggregation effect of AEE was performed using light transmission aggregometry in platelet-rich plasma (PRP). About 2 mL blood samples were collected and diluted by 3.8 % sodium citrate on the proportion of 9:1 in vacuum tubes, which were prepared for platelet aggregation analysis. Platelet aggregation was assessed in PRP, which was obtained by centrifugation of citrated whole blood at room temperature for 10 min at 1000 rpm. Then, PRP was placed into two cuvettes (0.25 mL PRP in each cuvette) and stirred with a rotor at 37 °C for 5 min, after which 5 μM ADP and 5 mM AA was added, respectively. The platelet-poor plasma (PPP) was obtained by centrifugation of PRP at room temperature for 10 min at 3000 rpm, which was used to set zero. Aggregation was measured with a Chrono-log Platelet Aggregometer Model 700 (Chrono-log Corp., USA). After the addition of an aggregating agent, light transmission at maximal aggregation was recorded. The results are expressed as percentage of maximal aggregation.

### Blood biochemical analysis

Blood samples of approximate 1 mL were collected into vacuum tubes without anticoagulant, and centrifuged at 4000 rpm for 10 min to obtain serum. Serum was analyzed using an XL-640 automatic biochemistry analyzer (Erba, German). Levels of total bilirubin (T-BIL), total protein (TP), albumin (ALB), alanine transaminase (ALT), aspartate aminotransferase (AST), alkaline phosphatase (ALP), gamma glutamyl transpeptidase (GGT), lactate dehydrogenase (LDH), blood urea nitrogen (BUN), creatinine (CR), glucose (GLU), glutamic pyruvic transaminase (GPT), creatine kinase (CK), calcium (Ca), phosphorus (P), triglycerides (TG) and total cholesterol (TCH) were analyzed in the experiment.

### Measurement of plasma TXB_2_ and 6-keto-PGF_1α_

Samples anticoagulated with EDTA-K_2_ were centrifuged at 1000 g for 10 min to obtain 200 μL of plasma for TXB_2_ and 6-keto-PGF_1α_ measurements. ELISA kit of TXB_2_ was purchased from U.S.A TSZ Biological Trade Co., Ltd. (New Jersey, USA) and 6-keto-PGF_1α_ ELISA kit was from Abcam Co., Ltd (Cambridge, UK). The ELISA operation was followed the protocols included with each kit.

### Statistical analysis

Statistical analyses were carried out using SPSS (Statistical Product and Service Solutions,IBM. Co., Armonk, NY, USA). Data obtained from experiment was expressed as mean ± standard deviation (SD). Statistical differences were evaluated by ANOVA with least significant difference (LSD) test. *P*-values less than 0.05 were considered to indicate statistical significance.

## Results

### Effects on hemorheology

Hemorheological parameters were analyzed by routine laboratory assays. The results are shown in Table 2. In comparison with the control group, whole blood viscosity and plasma viscosity were significantly increased in model group (*P* < 0.01, or *P* < 0.05). There was no statistical difference between CMC-Na and model groups. In order to eliminate the influence of the vehicle, drug-treated groups were compared with CMC-Na group. Aspirin reduced whole blood viscosity at low shear rate (*P* < 0.05), but did not influence whole blood viscosity at medium and high shear rates. Whole blood viscosity at low and medium shear rates in groups supplemented with AEE were significantly lower than that of CMC-Na group (*P* < 0.01). Whole blood viscosity at high shear rate was also significantly reduced by low-dose AEE (*P* < 0.05). Aspirin and eugenol remarkably reduced plasma viscosity (aspirin: *P* < 0.01; eugenol: *P* < 0.05). Low and high doses of AEE showed strong effects on reducing the plasma viscosity values (*P* < 0.01). No change was observed on whole blood viscosity and plasma viscosity between CMC-Na and combination groups.

**Table 2:** Drug effects on hemorheological parameters in k-carrageenan-induced rat tail thrombosis model (*n* = 10)

Groups	WBV (mPa.s)			PV (mPa.s)
	Low shear rate (5 s^−1^)	Medium shear rate (100 s^−1^)	High shear rate (200 s^−1^)	
Control	18.56 ± 3.03^##^	4.63 ± 0.77^##^	3.55 ± 0.79^##^	1.55 ± 0.28^#^
Model	27.75 ± 3.27	6.74 ± 0.78	5.24 ± 0.63	2.04 ± 0.47
CMC-Na	26.42 ± 2.66	6.87 ± 0.62	5.22 ± 0.52	1.97 ± 0.25
Aspirin	22.75 ± 3.57*	6.21 ± 0.62	5.21 ± 0.35	1.39 ± 0.13**
Eugenol	28.69 ± 2.93	6.87 ± 0.72	5.52 ± 0.79	1.63 ± 0.25*
AEE L	20.03 ± 2.75**	5.38 ± 0.78**	4.58 ± 0.67*	1.32 ± 0.12**
AEE M	22.60 ± 2.28**	5.48 ± 0.86**	4.74 ± 0.52	1.68 ± 0.61
AEE H	22.25 ± 3.60**	5.14 ± 0.69**	4.63 ± 0.62	1.50 ± 0.14**
Combination	27.45 ± 2.90	6.58 ± 1.06	5.28 ± 0.63	2.19 ± 0.30

After the last measurement of rat tail thrombosis length at 48 h, 0.8 mL of whole blood and 0.5 mL of plasma were collected for hemorheological parameter tests. Data are expressed as mean ± SD. ^#^ *P <* 0.05, ^##^ *P <* 0.01 compared with model group. *P < 0.05, **P < 0.01 compared with CMC-Na group. *WBV* whole blood viscosity, *PV* plasma viscosity. AEE L: AEE 18 mg/kg; AEE M: 36 mg/kg; AEE H: AEE 72 mg/kg; Combination: combination of aspirin and eugenol (molar ratio 1:1)

**Fig. 1:**
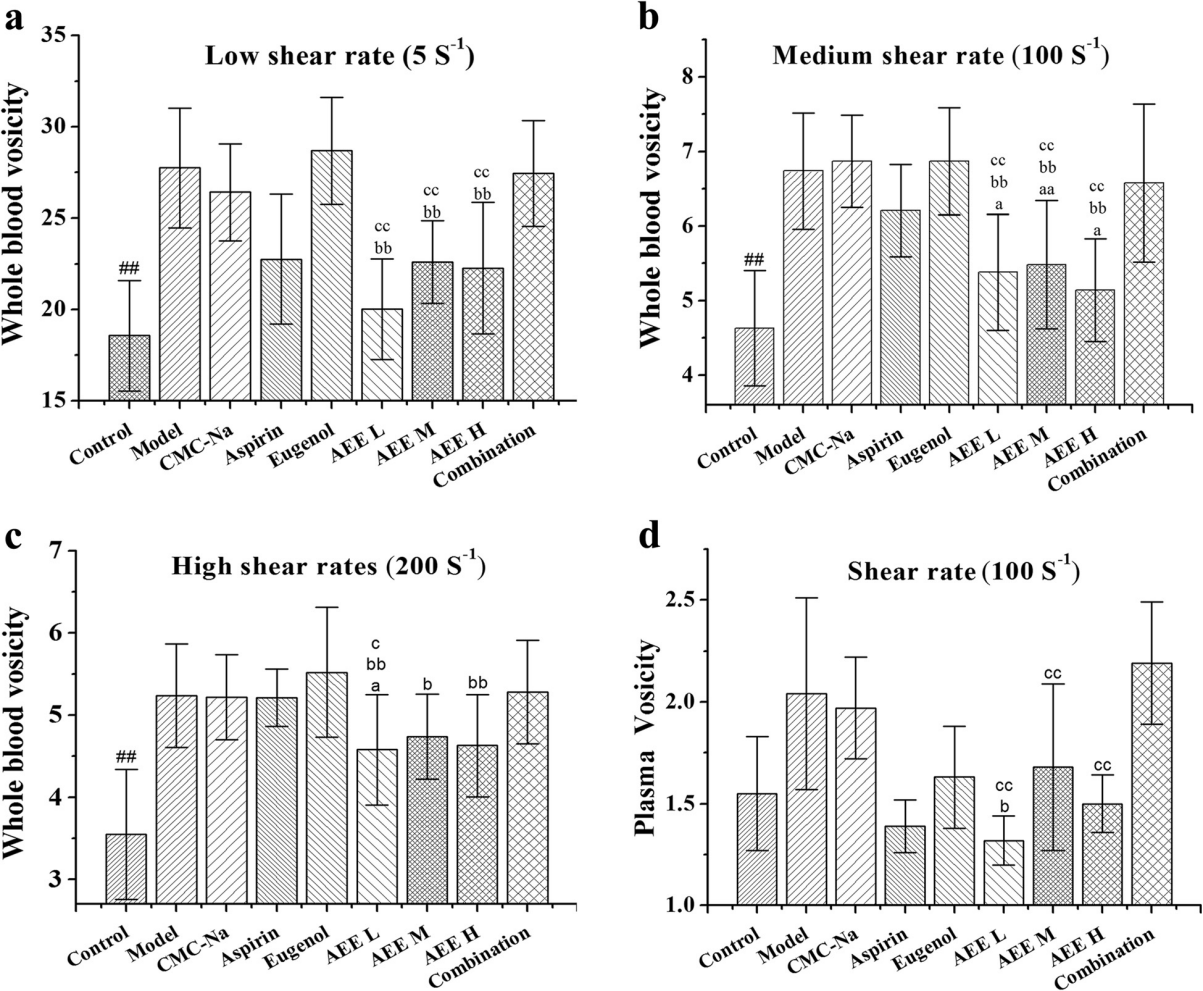
Comparative effects of aspirin, eugenol and AEE on hemorheological parameters (*n* = 10). (**a-c**): whole blood viscosity at the shear rates of 5 s^-1^, 100 s^-1^ and 200 s^-1^. (**d**): plasma viscosity at the shear rate of 100 s^-1^. ^#^ *P <* 0.05, ^##^ *P <* 0.01 compared with model group. ^a^ *P <* 0.05, ^aa^ *P <* 0.01 compared with aspirin group. ^b^ *P <* 0.05, ^bb^ *P <* 0.01 compared with eugenol group. ^c^ *P <* 0.05, ^cc^ *P <* 0.01 compared with combination group. Under the same molar quantity, medium dose of AEE showed better effects than its precursors on whole blood viscosity and plasma viscosity reduction in varying degrees. AEE L: AEE 18 mg/kg; AEE M: 36 mg/kg; AEE H: AEE 72 mg/kg. Combination: combination of aspirin and eugenol (molar ratio 1:1)

In order to find out the difference in results between AEE and its precursors, multiple comparisons were carried out. In the experiment, the molar quantities of aspirin, eugenol and medium-dose AEE were same. The results are shown in Fig. 1. AEE showed stronger effects on whole blood viscosity reduction at low shear rate than eugenol and combination groups (*P* < 0.01, Fig. 1a). In comparison with aspirin, eugenol and the combination groups, AEE significantly reduced whole blood viscosity at medium and high shear rates (*P* < 0.01 or *P* < 0.05 Fig. 1b and c). Interestingly, the plasma viscosity values of each AEE group was significantly lower than that of combination group (*P* < 0.01, Fig. 1d). Moreover, the mean values of whole blood viscosity and plasma viscosity in AEE groups were lower than other drug-treated groups. Medium-dose AEE reduced whole blood viscosity more effectively than eugenol (*P* < 0.01 or *P <* 0.05 Fig. 1a, b and c). Moreover, medium-dose AEE showed better effects than aspirin on whole blood viscosity at medium shear rate (*P* < 0.01 Fig. 1b). There were significant differences on plasma viscosity and whole blood viscosity (at low and medium shear rates) between AEE M and combination groups (*P* < 0.01 Fig. 1a, b and d). To varying degrees, under the equimolar quantity of drugs, medium-dose AEE stronger reduced whole blood viscosity and plasma viscosity than aspirin, eugenol and combination of them from the different hemorheological parameters.

### Effect of AEE on platelet aggregation in vivo

The results of platelet aggregation are shown in Table 3. Platelet aggregation induced by AA and ADP was significantly increased in model group (*P* < 0.01). No difference was observed between model and CMC-Na groups. AEE, eugenol and aspirin remarkably reduced AA-induced platelet aggregation than CMC-Na in varying degrees (*P* < 0.05 or *P* < 0.01). The mean values in aspirin and eugenol groups were 20.44 and 27.10, which indicated aspirin had stronger effects than eugenol on inhibiting AA-induced platelet aggregation. In regard to ADP-induced platelet aggregation, the values in aspirin, combination, medium and high-dose AEE groups were lower than CMC-Na group (*P* < 0.01).

**Table 3:** Effect of AEE on platelet aggregation in k-carrageenan-induced rat tail thrombosis model (*n* = 10)

Groups	AA-induced PAg	ADP-induced PAg
Control	8.70 ± 1.34^##^	31.90 ± 2.77^##^
Model	29.00 ± 1.49	50.10 ± 3.34
CMC-Na	30.10 ± 2.13	51.90 ± 2.28
Aspirin	20.44 ± 2.74**	40.89 ± 4.26**
Eugenol	27.10 ± 3.14*	50.10 ± 2.23
AEE L	18.70 ± 3.49**	49.30 ± 2.79
AEE M	11.80 ± 3.70**	43.30 ± 3.13**
AEE H	15.70 ± 3.43**	42.20 ± 3.26**
Combination	11.50 ± 2.50**	45.50 ± 3.17**

5 μM ADP and 5 mM AA were added separately to platelet-rich plasma (PRP), which was used to assess platelet aggregation. Results are expressed as the percentage of maximal aggregation. Data are expressed as mean ± SD. ^#^ *P <* 0.05, ^##^ *P <* 0.01 compared with the model group. *P < 0.05, **P < 0.01 compared with the CMC-Na group. PAg: platelet aggregation. AEE L: AEE 18 mg/kg; AEE M: 36 mg/kg; AEE H: AEE 72 mg/kg; Combination: combination of aspirin and eugenol (molar ratio 1:1)

**Fig. 2:**
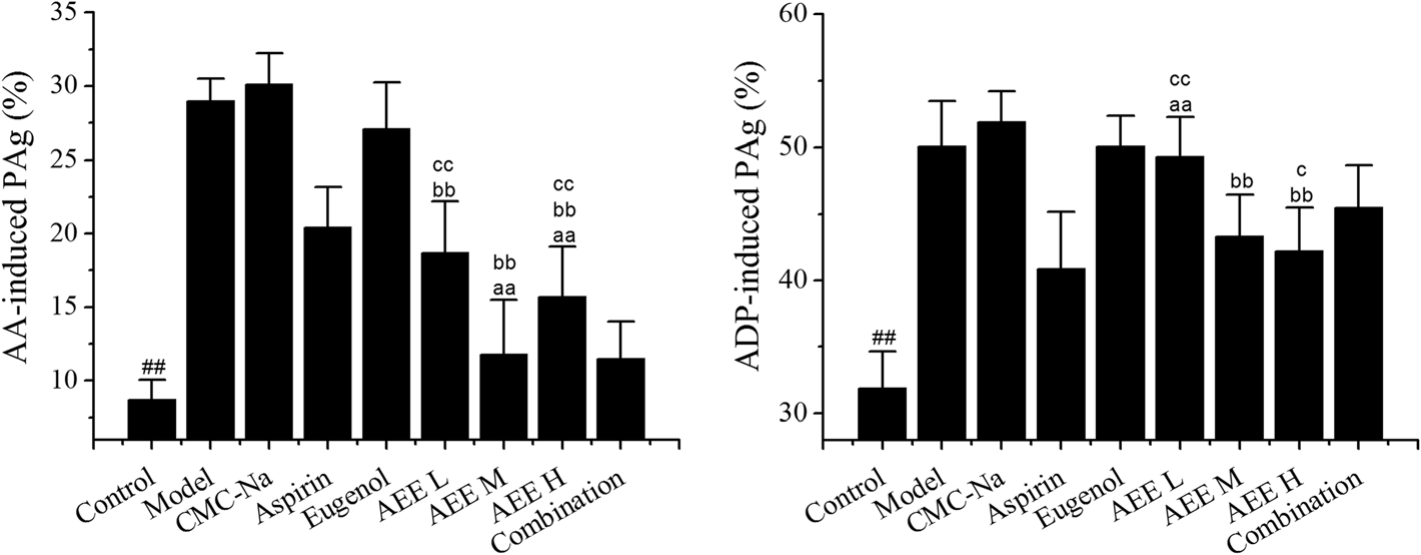
Comparative effects of aspirin, eugenol and AEE on platelet aggregation in k-carrageenan-induced rat tail thrombosis model (*n* = 10). ^#^ *P <* 0.05, ^##^ *P <* 0.01 compared with model group. ^a^ *P <* 0.05, ^aa^ *P <* 0.01 compared with aspirin group. ^b^ *P <* 0.05, ^bb^ *P <* 0.01 compared with eugenol group. ^c^ *P <* 0.05, ^cc^ *P <* 0.01 compared with combination group. No difference was observed between model and CMC-Na groups, which indicated that CMC-Na made no influence on PAg index. AEE showed strong effects than aspirin and eugenol at the same molar quantity. PAg:platelet aggregation. AEE L: AEE 18 mg/kg; AEE M: 36 mg/kg; AEE H: AEE 72 mg/kg; Combination: combination of aspirin and eugenol (molar ratio 1:1)

The effects of different drug on platelet aggregation were evaluated in the study (seen in Fig. 2). Medium- and high-dose AEE showed stronger effects than aspirin on reducing AA-induced platelet aggregation values (*P* < 0.01). Meanwhile, three different doses of AEE displayed better effects than eugenol (*P* < 0.01). ADP-induced platelet aggregation values in low-dose AEE group were higher than aspirin and combination groups (*P* < 0.01). Medium- and high-dose AEE significantly inhibited ADP-induced platelet aggregation more effectively than eugenol (*P* < 0.01). The values of ADP-induced platelet aggregation in high-dose AEE group were lower than that in combination group (*P* < 0.05). Based on these results, under the same molar quantity, medium-dose AEE showed better effects than aspirin and eugenol on AA-induced platelet aggregation and possessed more effective effects of inhibiting ADP-induced platelet aggregation than eugenol.

### Blood biochemical results

The results of blood biochemistry are given in Table 4. In comparison with control group, TP, ALB, ALP, ALT, Ca and TG in model group were significantly reduced (*P* < 0.01), AST and TCH were remarkably elevated (*P* < 0.05 and *P* < 0.01). The results of ALB, ALT and LDH between model and CMC-Na groups showed some differences (*P* < 0.05, or *P* < 0.01). When compared with CMC-Na group, aspirin significantly reduced the levels of T-BIL, AST, LDH, TG and TCH, and elevated the levels of TP, ALB, BUN and Ca (*P* < 0.05, or *P* < 0.01). In addition, there was no significant difference between CMC-Na and eugenol groups.

**Table 4:** Biochemical parameters in rats intragastrically administered different drugs (*n* = 10)

Variables	Unit	Control	Model	CMC-Na	Aspirin	Eugenol	AEE L	AEE M	AEE H	Combination
T-BIL	umol/L	0.90 ± 0.13	0.97 ± 0.19	1.00 ± 0.17	0.74 ± 0.12**	1.11 ± 0.17	0.72 ± 0.15**	0.65 ± 0.10**	0.86 ± 0.12	1.17 ± 0.17*
TP	g/L	58 ± 2.9^##^	53 ± 4.3	50 ± 3.4	60 ± 2.2**	50 ± 4.7	59 ± 2.4**	60 ± 3.3**	58 ± 4.1**	61 ± 3.9**
ALB	g/L	23 ± 1.0^##^	20 ± 1.4	18 ± 1.1^##^	20 ± 0.8*	19 ± 1.4	21 ± 2.3**	21 ± 0.7**	20 ± 1.1*	22 ± 1.2**
ALT	U/L	69 ± 10^##^	57 ± 9	67 ± 12^#^	61 ± 5	68 ± 13	65 ± 6	65 ± 6	58 ± 14	67 ± 13
AST	U/L	79 ± 11^#^	93 ± 10	85 ± 12	65 ± 8**	96 ± 14	76 ± 10	75 ± 16	74 ± 12	120 ± 14**
ALP	U/L	240 ± 19^##^	145 ± 22	156 ± 22	178 ± 27	137 ± 23	180 ± 25	181 ± 21	177 ± 11	172 ± 54
GGT	U/L	3.16 ± 0.49	3.34 ± 0.44	3.20 ± 0.25	3.44 ± 0.45	3.15 ± 0.67	3.55 ± 0.57	3.72 ± 0.64	3.61 ± 0.51	3.62 ± 0.60
LDH	U/L	141 ± 28	129 ± 29	161 ± 18^#^	123 ± 31*	190 ± 27	128 ± 20	126 ± 22	140 ± 38	227 ± 36**
BUN	mmol/L	8.0 ± 0.6	8.9 ± 1.7	9.9 ± 1.7	11.5 ± 1.4*	10.3 ± 1.9	9.4 ± 0.7	9.7 ± 1.5	8.3 ± 0.9 *	12.5 ± 1.3**
CR	umol/L	27.3 ± 4.6	29.3 ± 4.9	29.0 ± 4.8	37.4 ± 3.6*	31.6 ± 4.1	24.9 ± 3.8	32.1 ± 4.6	26.2 ± 3.2	36.4 ± 4.7**
Glu	mmol/L	8.01 ± 0.68	8.11 ± 0.68	7.98 ± 0.53	7.78 ± 0.38	7.52 ± 0.30	7.50 ± 0.81	7.40 ± 0.25	7.57 ± 0.62	7.90 ± 0.81
Ca	mmol/L	2.42 ± 0.06^##^	2.08 ± 0.10	2.12 ± 0.15	2.30 ± 0.05**	2.12 ± 0.12	2.47 ± 0.12**	2.35 ± 0.05**	2.24 ± 0.14*	2.19 ± 0.06
P	mmol/L	3.31 ± 0.19	3.47 ± 0.25	3.50 ± 0.60	3.36 ± 0.40	3.64 ± 0.43	3.23 ± 0.45	3.36 ± 0.20	3.54 ± 0.15	3.53 ± 0.22
TG	mmol/L	1.19 ± 0.21^##^	0.66 ± 0.09	0.78 ± 0.06	0.56 ± 0.09*	0.69 ± 0.16	0.71 ± 0.19	0.59 ± 0.15	0.58 ± 0.27*	0.77 ± 0.21
TCH	mmol/L	1.37 ± 0.07^##^	1.98 ± 0.08	1.81 ± 0.23	1.53 ± 0.17**	1.93 ± 0.18	1.62 ± 0.12*	1.56 ± 0.18*	1.55 ± 0.26**	1.96 ± 0.29

After the last measurement of thrombosis length at 48 h, blood was drawn from the heart. Serum samples were prepared by centrifugation and analyzed using an automatic biochemistry analyzer. ^#^ *P* < 0.05, ^##^ *P* < 0.01 compared with the model group. *P < 0.05, **P < 0.01 compared with the CMC-Na group. AEE L: AEE 18 mg/kg; AEE M: 36 mg/kg; AEE H: AEE 72 mg/kg; Combination: combination of aspirin and eugenol (molar ratio 1:1)

In comparison with CMC-Na group, the levels of ALB and Ca were significantly increased in AEE groups (*P* < 0.05, or *P* < 0.01), whereas TCH was significantly reduced (*P* < 0.05, or *P* < 0.01). Low- and medium-dose AEE significantly increased the level of TP and reduced the level of T-BIL (*P* < 0.01). The TG and BUN levels in high-dose AEE group were significantly reduced (*P* < 0.05). In comparison with the CMC-Na group, the levels of ALB, AST, LDH and BUN in combination group were significantly elevated (*P* < 0.01). The differences in blood biochemical indexes among the groups treated with three doses of AEE were dose-independent.

### Effects of AEE on TXB_2_ and 6-keto-PGF_1α_

The results of TXB_2_ and 6-keto-PGF_1a_ are shown in Table 5. TXB_2_ and TXB_2_/6-keto-PGF_1α_ ratio were elevated in model group in comparison with those in control group, but 6-keto-PGF_1α_ was declined (*P* < 0.01). There was no difference in TXB_2_ between CMC-Na and model groups. However, a significant decrease of 6-keto-PGF_1α_ and an increase of TXB_2_/6-keto-PGF_1α_ ratio were observed (*P* < 0.01). Eugenol and aspirin significantly increased 6-keto-PGF_1α_ and lowered TXB_2_/6-keto-PGF_1α_ ratio than CMC-Na (*P* < 0.01). In comparison with CMC-Na group, low-, medium- and high-dose AEE had significantly increased 6-keto-PGF_1α_ and reduced TXB_2_ with a drop of TXB_2_/6-keto-PGF_1α_ ratio (*P* < 0.01). No dose response relationship was observed in AEE groups on TXB_2_ and 6-keto-PGF_1α_.

**Table 5:** Effects of each treatment on TXB_2_, 6-keto-PGF_1α_ and the TXB_2_/6-keto-PGF_1a_ ratio in k-carrageenan-induced rat tail thrombosis model (*n* = 10)

Groups	TXB_2_	6-keto-PGF_lα_	TXB_2_/6-keto-PGF_lα_
Control	498 ± 32^##^	889 ± 28^##^	0.56 ± 0.04^##^
Model	696 ± 39	586 ± 24	1.19 ± 0.08
CMC-Na	703 ± 37	550 ± 22^##^	1.28 ± 0.08^##^
Aspirin	658 ± 44*	741 ± 23**	0.89 ± 0.06**
Eugenol	678 ± 58	699 ± 30**	0.97 ± 0.09**
AEE L	605 ± 22**	667 ± 17**	0.91 ± 0.04**
AEE M	612 ± 45**	737 ± 13**	0.83 ± 0.07**
AEE H	644 ± 23**	599 ± 26**	1.08 ± 0.07**
Combination	598 ± 51**	701 ± 31**	0.86 ± 0.10**

Blood samples anticoagulated with EDTA-K_2_ were centrifuged at 1000 g for 10 min to obtain plasma, which was analyzed using ELISA kits. Data are expressed as mean ± SD. ^##^ *P* < 0.01 compared with the model group. *P < 0.05, **P < 0.01 compared with the CMC-Na group. AEE L: AEE 18 mg/kg; AEE M: 36 mg/kg; AEE H: AEE 72 mg/kg; Combination: combination of aspirin and eugenol (molar ratio 1:1)

**Fig. 3:**
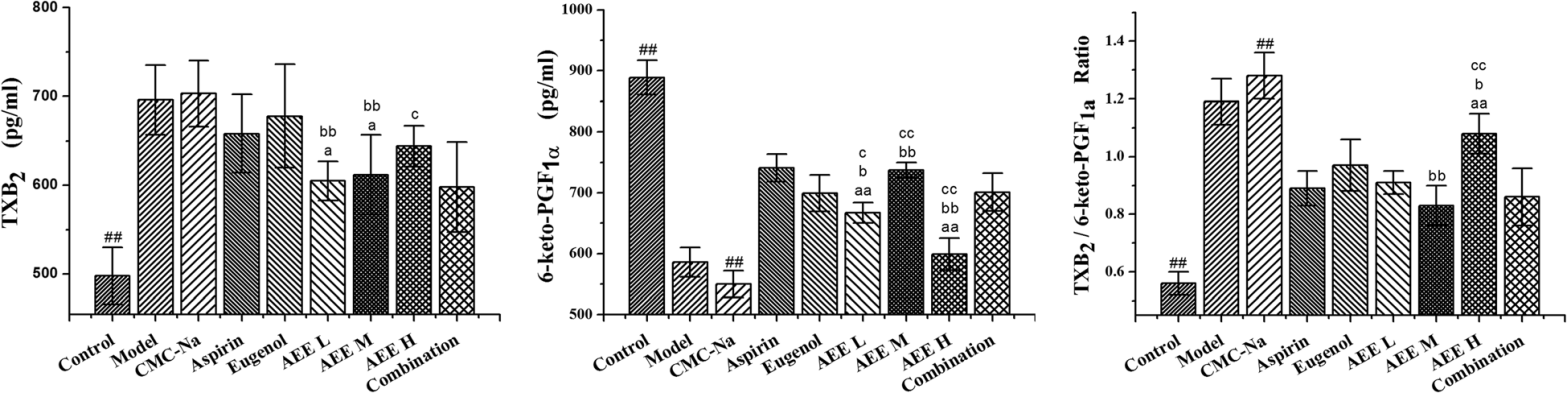
Comparative effects of aspirin, eugenol and AEE on TXB_2_, 6-keto-PGF_1α_ and TXB_2_/6-keto-PGF_1a_ ratio in different groups (*n* = 10). ^#^ *P <* 0.05, ^##^ *P <* 0.01 compared with model group. ^a^ *P <* 0.05, ^aa^ *P <* 0.01 compared with aspirin group. ^b^ *P <* 0.05, ^bb^ *P <* 0.01 compared with eugenol group. ^c^ *P <* 0.05, ^cc^ *P <* 0.01 compared with combination group. AEE L: AEE 18 mg/kg; AEE M: 36 mg/kg; AEE H: AEE 72 mg/kg; Combination: combination of aspirin and eugenol (molar ratio 1:1)

Figure 3 shows the effects different drugs on TXB_2_ and 6-keto-PGF_1α._ In comparison with aspirin and eugenol, AEE reduced TXB_2_ and 6-keto-PGF_1α_ to varying degrees with the exception of 6-keto-PGF_1α_ in medium-dose AEE group. TXB_2_ in high-dose AEE group were higher than combination group (*P* < 0.05). Three different doses of AEE remarkably lowered 6-keto-PGF_1α_ in comparison with combination group (*P* < 0.05 or *P* < 0.01). Under the same molar quantity, medium-dose AEE significantly reduced TXB_2_ than aspirin and eugenol (*P* < 0.05 or *P* < 0.01). Results of 6-keto-PGF_1α_ in medium-dose AEE group were higher than eugenol and combination groups. Meanwhile, the TXB_2_/6-keto-PGF_1α_ ratio in medium-dose AEE group was significantly lower than that in eugenol (*P* < 0.01). Therefore, equimolar AEE possessed better effects on reducing TXB_2_, increasing 6-keto-PGF_1α_ and regulation of TXB_2_/6-keto-PGF_1α_ ratio than its precursors.

## Discussion

Several chemical drugs are used to prevent or treat thrombosis such as clopidogrel, ticlopidine, dabigatran and apixaban. However, available antithrombosis agents still have some limitations such as gastrointestinal damage and hemorrhage. Therefore, there is an obvious need for more efficacious and alternative treatment options for thrombosis. A previous study showed that AEE could reduce platelet aggregation in vitro, fibrinogen concentration and regulate coagulation parameters, indicating that AEE was a promising drug candidate for preventing cardiovascular diseases [23]. It was important to characterize its possible mechanism from different views.

Blood hemorheology is an integrated branch of physics and medicine. Increased blood viscosity is a risk factor for thrombosis, atherosclerosis and other cardiovascular events [24]. The increased blood and plasma viscosity in model group can be related to the acute inflammation caused by k-carrageenan injection [23, 25]. AEE decreased whole blood and plasma viscosity, which contributed to ameliorating blood circulation. It also proved that AEE could reduce fibrinogen concentration and normalize blood components [23]. Changes of blood components, especially the reduction of hematocrit, may be responsible for the decrease of viscosity. Additionally, blood viscosity is also determined by shear rate, red blood cell deformability and aggregation. The effects of AEE and its dosage influence on hemorheological parameters should be assessed in the future studies. Some differences were observed between aspirin and combination groups in hemorheological parameters. The mean values of plasma component such as TP, ALB, TG and TCH in aspirin group were lower than those in combination group, which may be the reason for the decrease in plasma viscosity. It was speculated that the differences in RBC deformability and aggregation in aspirin and combination groups were responsible for the different results in whole blood viscosity.

Salicylic acid is the major metabolite of AEE confirmed by in vivo and in vitro experiments [26]. As a classic drug, aspirin is used therapeutically in the prevention of cardiovascular disease by blocking TXA_2_ synthesis. Salicylic acid, also the main metabolite of aspirin, is the primary substance responsible for pharmacological function of aspirin on platelet aggregation. AEE and aspirin share the same metabolite as salicylic acid; therefore, AEE and aspirin likely inhibit platelet aggregation via similar mechanism. The effects of eugenol on human platelet aggregation had been investigated and proved that eugenol was a strong platelet aggregation inhibitor [12, 13]. Vascular endothelial cell damage caused by k-carrageenan may be the reason for the increased platelet aggregation in model group [18]. The vehicle, CMC-Na, had no effect on platelet aggregation. In the case of equal molar quantity, AEE inhibited the platelet aggregation and showed stronger effects than eugenol on AA and ADP-induced platelet aggregation, and better effects than aspirin on AA-induced platelet aggregation. Based on the results, the effect of AEE on platelet aggregation is likely produced by both salicylic acid and eugenol. AEE is decomposed into salicylic acid and eugenol by the enzyme after absorption, after which salicylic acid and eugenol as the major metabolites showed their original activities and acted synergistically to increase the inhibitory effect on platelet aggregation. Previous studies have confirmed that platelet aggregation is an important influence in blood viscosity [27]. Thus, the reduced platelet aggregation caused by AEE was another factor contributing to the decreases of blood and plasma viscosity.

In order to explore the further mechanisms of inhibiting platelet aggregation, the levels of TXB_2_ and 6-keto-PGF_1α_ in plasma were measured. TXB_2_ and 6-keto-PGF_1α_ are the stable hydrolysis products of thromboxane A2 (TXA_2_) and prostacyclin I2 (PGI_2_), respectively. TXA_2_ is a potent inducer of platelet aggregation and vasoconstriction [6]. PGI_2_ is a powerful vasodilator that inhibits platelet aggregation [28]. Arachidonate is a precursor of both PGI_2_ and TXA_2_. Cyclooxygenase (COX) plays an important role in arachidonate metabolic pathways to generate PGI_2_ and TXA_2_ [29]. Some evidences have demonstrated that both aspirin and eugenol inhibit COX activity [30–32]. Aspirin, as an inhibitor of COX, could reduce both the level of TXB_2_ and 6-keto-PGF_1α_. However, the level of 6-keto-PGF_1α_ in aspirin group was higher than that of CMC-Na group. The previous study showed that aspirin significantly decreased the length of rat tail thrombosis [23]. Thrombosis formation may produce the reductive effects on 6-keto-PGF_1α_. Aspirin inhibits the thrombosis formation in the rat tail, and then weakens the decreasing effects caused by k-carrageenan on 6-keto-PGF_1α_. Suppression of inflammation by aspirin may be the reason of reducing rat tail thrombosis length [33]. Platelets primarily process PGH_2_ to TXA_2_ and vascular endothelial cells primarily process PGH_2_ to PGI_2_. Aspirin selectively inhibits the production of TXA_2_ in platelet, while sparing endothelial PGI_2_ synthesis [34]. New cyclooxygenase could be produced in endothelial cells, but cannot in anucleate platelets. It was speculated that the different effects of aspirin on platelets and endothelial cells and the newly produced cyclooxygenase from endothelial cells may be the reasons for the raised 6-keto-PGF_1α_ in aspirin group. Under equal molar quantity, AEE produced better effects than single using of eugenol or aspirin on TXB_2_ and 6-keto-PGF_1α_. According to these results, AEE might significantly down-regulate TXB_2_ and up-regulate 6-keto-PGF_1α_ with the TXB_2_/6-keto-PGF_1α_ decreased, indicating that the antithrombotic action of AEE was associated with the regulation of TXB_2_ and 6-keto-PGF_1α_.

Analysis of blood biochemical parameters could help veterinarians and breeders assess the general health status of animals [35, 36]. Injection of k-carrageenan disturbed the blood biochemical profile through the increase of AST and TCH and the reduction of TP, ALB, ALP, ALT, Ca and TG in model group. These alterations may be related to the stress caused by acute inflammation. Previous study showed that leukocyte and monocyte counts were significantly increased after k-carrageenan injection, which indicated that the rats suffered from acute and systemic inflammation [23]. Intense inflammation could cause the disorder of liver function, and then result in the changes of biochemical profile [37, 38]. In the present study, the reduction of TP, ALB, ALP and the increasing of AST in model group indicated abnormal liver function. Ca, a key factor in thrombosis formation, may be depleted in thrombosis formation process, which may be the reason for the reduction of Ca in model group. The increase of TCH and the reduction of TG may be related to rat body response [39]. It has been proved that AEE could regulate blood lipids in hyperlipidemic rats [40]. The results of TG and TCH indexes in this experiment were similar as found in previous studies [16, 40]. AEE showed positive effects on changing biochemical characters and possessed the ability to normalize the biochemical profile following inflammation, which may be supported by the prolonged time of drug action [41]. In the present study, the blood biochemical results in eugenol group showed no differences in comparison with CMC-Na group. Several reasons may be for these results. Blood biochemical parameters were drastically changed by k-carrageenan injection, which may mask the changes caused by eugenol. Moreover, the dosage of eugenol used in the experiment or eugenol itself was unable to affect biochemical parameters. Previous studies indicated that administration of 150 mg eugenol for 7 days had no significant effects on clinical biochemical parameters in humans [42].

CMC-Na is a reliable drug carrier that is used in a wide range of applications in the pharmaceutical industry [43, 44]. In this study, CMC-Na as vehicle had influence on 6-keto-PGF_lα_, TXB_2_/6-keto-PGF_1a_ ratio, ALB, ALT and LDH. In order to demonstrate the activity of AEE, the comparisons were carried out between CMC-Na and treated groups. Therefore, the effect of CMC-Na was eliminated. Tween 80 is widely applied in emulsifying and dispersing substances in medicinal products. In the present study, Tween 80 was used to prepare eugenol. It had been confirmed that the body had a great tolerance to Tween 80 [45]. The effects of Tween 80 and combination of CMC-Na and Tween 80 were not investigated in this experiment.

AEE on the design of prodrug principle contains ester bond structure that is decomposed easily. Pharmacokinetics studies showed that the plasma concentration of AEE itself was extremely low; indeed it was not detectable in plasma. Therefore, it is speculated that the degradation products of AEE such as salicylic acid and eugenol are responsible for its effects. Salicylic acid and eugenol may play an efficient role and interact in a synergistic manner to prevent thrombosis formation. More studies should be conducted to investigate the action mechanism of AEE on thrombosis prevention. In addition, as a promising chemical compound, AEE should be studied in preclinical experiments to assess its therapeutic effects in various species.

## Conclusion

The results obtained in our study showed that AEE had potent antithrombotic effects in rats with k-carrageenan-induced tail thrombosis. Moreover, AEE showed better antithrombotic effect than its precursors under same molar quantity. From the findings, the preventive effect of AEE may come from antiplatelet aggregation, reducing blood and plasma viscosity, balancing TXB_2_/6-keto-PGF_1a_ ratio and normalizing blood biochemical parameters. These therapeutic effects of AEE may be related to the synergetic actions of aspirin and eugenol. It may be concluded that AEE was a good candidate for antithrombotic agent. These findings provide new insight into the action mechanism of AEE on preventing thrombosis. More studies are necessary to investigate its mechanism of action such as the protective effects of vascular endothelial cells, the way on anti-platelet aggregation and the influences on metabolic profile.

## Abbreviations

6-keto-PGF_1α_, 6-keto prostaglandin F_1α_; AA, arachidonic acid; ADP, adenosine diphosphate; AEE, aspirin eugenol ester; ALB, albumin; ALP, alkaline phosphatase; ALT, alanine transaminase; AST, aspartate aminotransferase; BUN, blood urea nitrogen; Ca, calcium; CK, creatine kinase; CMC-Na, sodium carboxymethyl cellulose; CR, creatinine; GGT, gamma glutamyl transpeptidase; GLU, glucose; GPT, glutamic pyruvic transaminase; LDH, lactate dehydrogenase; P, phosphorus; PGI_2_, prostacyclin I2; PPP, platelet-poor plasma; PRP, platelet-rich plasma; T-BIL, total bilirubin; TCH, total cholesterol; TG, triglycerides; TP, total protein; TXA_2_, thromboxane A2; TXB_2_, thromboxane B_2_.
